# Prävention des ischämischen Schlaganfalls im Alter

**DOI:** 10.1007/s00391-024-02336-x

**Published:** 2024-08-06

**Authors:** Bernhard Iglseder, J. Sebastian Mutzenbach

**Affiliations:** 1 Universitätsklinik für Geriatrie der PMU, Uniklinikum Salzburg – Campus Christian-Doppler-Klinik, Ignaz-Harrer-Straße 79, 5020 Salzburg, Österreich; 2 Universitätsklinik für Neurologie, neurologische Intensivmedizin und Neurorehabilitation, Uniklinikum Salzburg – Campus Christian-Doppler-Klinik, Salzburg, Österreich

**Keywords:** Zerebrovaskuläre Erkrankungen, Risikofaktoren, Primärprävention, Sekundärprävention, Hochbetagte, Cerebrovascular diseases, Risk factors, Primary prevention, Secondary prevention, Aged, 80 and over

## Abstract

Der Schlaganfall ist eine der Hauptursachen für bleibende Behinderung und Tod; das Risiko steigt mit dem Alter. Der Primär- und Sekundärprävention kommt eine hohe Priorität zu. Die Behandlung von Risikofaktoren wie Bluthochdruck, Diabetes mellitus und Hyperlipidämie ist neben der Optimierung von Lebensstil und Ernährung ebenso bedeutend wie die Antikoagulation bei Vorhofflimmern. In der Rezidivprophylaxe spielen Thrombozytenfunktionshemmer eine Rolle, Karotisoperation oder Stenting kommen bei ausgewählten Individuen zum Einsatz. Für alte Menschen gibt es nur geringe Studienevidenz; eine individualisierte Therapieplanung berücksichtigt funktionellen Status und Komorbiditäten.

## Abstract (en)

Stroke is one of the main causes of permanent disability and death and the risk increases with age. Primary and secondary prevention therefore have a high priority. The treatment of risk factors, such as high blood pressure, diabetes mellitus and hyperlipidemia is just as important as anticoagulation in atrial fibrillation, in addition to optimization of lifestyle and diet. Platelet function inhibitors play a role in the prophylaxis of recurrence, carotid surgery and stenting are used in selected patients. There is little study evidence for old people, individualized treatment planning takes functional status and comorbidities into account.

## Lernziele

Nach der Lektüre dieses Beitrags kennen Sie

- die wesentlichen Risikofaktoren für Schlaganfälle im Alter.
- die unklare Studienlage bei multimorbiden Hochbetagten.
- die Notwendigkeit einer individualisierten Therapieplanung.
- Zielwerte für Hypertonie und Hyperlipämie bei gesunden alten Menschen.
- Nutzen und Risiken der oralen Antikoagulation bei Vorhofflimmern (VHF).

## Einleitung

Etwa 30 % der Schlaganfälle (SA) treten jenseits des 80. Lebensjahres auf, der SA ist eine häufige Erkrankung des alten Menschen [1]. Mit dem Alter steigt die Zahl **kardioembolischer Schlaganfälle**

kardioembolischer Schlaganfälle,

in erster Linie bedingt durch VHF. Auch **hämorrhagische Schlaganfälle**

hämorrhagische Schlaganfälle

sind im Alter häufiger, wobei Amyloidangiopathie und Antikoagulation Ursachen mit Altersbezug darstellen.

Der SA ist eine der Hauptursachen für **bleibende Behinderung**

bleibende Behinderung,

daher kommt aufgrund der Demografie der Prävention enorme Bedeutung zu. Die Datenlage ist jedoch komplex: Alte Menschen, insbesondere jenseits des 80. Lebensjahres, sind in klinischen Studien unterrepräsentiert, daher ist das evidenzbasierte Wissen für diese Kollektive unvollständig [2].

### Merke

- Das evidenzbasierte Wissen zur Therapie alter Menschen ist limitiert.
- Dies gilt in besonderem Maß bei Vorliegen von Frailty und Multimorbidität.

In der Altersgruppe ≥ 80 Jahre sind etwa 20 % von Frailty (Gebrechlichkeitssyndrom) betroffen. Hier kann das strikte Vorgehen nach Leitlinien, die Studiendaten aus jüngeren Kollektiven repräsentieren, mit negativen Folgen behaftet sein. Polymedikation, Sturzrisiko und Frailty müssen mit präventiven Maßnahmen in Einklang gebracht werden; eine individualisierte Therapieplanung sichert die praktische Umsetzbarkeit.

In dieser Übersicht werden Herangehensweisen für fitte alte Menschen dargestellt, aber auch Möglichkeiten für gebrechliche, kognitiv oder funktionell Beeinträchtigte skizziert. Etablierte Empfehlungen zur **Lebensstilmodifikation**

Lebensstilmodifikation

gelten auch im Alter [3].

### Fallbeispiel

Eine 84-jährige Frau wird mit ungerichtetem Schwindel und Palpitationen in die Notaufnahme gebracht. Sie lebt selbstversorgend mit dem Gatten; anamnestisch lassen sich ein behandelter Hypertonus, eine behandelte Osteoporose, ein als geheilt anzusehendes Mammakarzinom sowie eine Hüftendprothese bei Koxarthrose erheben. Die Untersuchungen ergeben ein tachykardes VHF, hypertensive Blutdruckwerte (190/90 mm Hg) sowie erhöhte Blutfette (Low-Density-Lipoprotein[LDL]-Cholesterin 160 mg/dl). Nach der Blutdrucksenkung bessert sich die Symptomatik. Die Echokardiographie zeigt eine gute Pumpfunktion und weitgehend intakte Klappen; in der Karotissonographie finden sich beidseits einzelne flache echoreiche Bifurkationsplaques. In Absprache mit der Patientin wird eine orale Antikoagulation mit einem Faktor-Xa-Hemmer eingeleitet. Die vorbestehende antihypertensive Therapie mit einem Kalziumantagonisten wird gesteigert und um einen β‑Blocker zur Frequenzkontrolle erweitert. Auf eine Statintherapie wird verzichtet, da Muskelschmerzen bei einem früheren Therapieversuch berichtet werden.

## Primärprävention

Zur Schlaganfallprävention im Alter stehen die Behandlung konventioneller **Arterioskleroserisikofaktoren**

Arterioskleroserisikofaktoren

und die Antikoagulation bei VHF im Vordergrund.

### Hypertonie

Bluthochdruck ist der Hauptrisikofaktor für SA; randomisierte kontrollierte Studien zeigten eine deutliche Risikoreduktion durch die Therapie [4]. Eine Metaanalyse legt nahe, dass es keine relevante Altersabhängigkeit gibt [5]. Eine Schlüsselstudie zur Blutdruckbehandlung im Alter, HYVET, untersuchte 3845 Menschen mit Werten des systolischen Blutdrucks > 160 mm Hg. Primäre Endpunkte waren fataler oder nichtfataler SA; die Studie wurde beendet, da die Interventionsgruppe (**Indapamid**

Indapamid,

falls nötig ergänzt um **Perindopril**

Perindopril)

statistisch signifikant niedrigere Ereignisraten und Todesfälle aufwies. Auch **Herzinsuffizienz**

Herzinsuffizienz

und andere kardiovaskuläre Ereignisse waren in der Interventionsgruppe seltener. Die Studie repräsentiert eine weitgehend gesunde Population. Ob die Ergebnisse vollinhaltlich auf gebrechliche alte Menschen übertragen werden können, ist nicht eindeutig zu beantworten. In einer Subanalyse fanden sich keine Hinweise auf eine Wechselwirkung zwischen dem Effekt der Behandlung des Bluthochdrucks und dem Vorhandensein von Frailty zu Studienbeginn. Sowohl die frailen als auch die fitten Älteren schienen von der antihypertensiven Therapie zu profitieren [6]. Auch die SPRINT-Studie untersuchte eine relevante Zahl weitgehend gesunder Teilnehmer*innen jenseits des 75. Lebensjahres; wie in HYVET fanden sich positive Behandlungsergebnisse auch bei den weniger gesunden Personen [7]. Aktuell wird ein Therapiebeginn bei weitgehend gesunden alten Menschen ab Werten des systolischen Blutdrucks > 150 mm Hg empfohlen.

#### Merke

Bei weitgehend gesunden alten Menschen wird eine antihypertensive Therapie bei Werten des systolischen Blutdrucks > 150 mm Hg empfohlen.

Für gebrechliche alte Menschen gibt es keine verlässlichen Daten zu Zielwerten, ein medikamentös behandelter systolischer Blutdruck < 130 mm Hg ist allerdings mit einer ungünstigen Prognose quoad vitam vergesellschaftet [8]. In Alterskollektiven ist also eine **individualisierte Entscheidungsfindung**

individualisierte Entscheidungsfindung

nötig: Aufmerksamkeit verdient die **orthostatische Hypotension**

orthostatische Hypotension,

die zu einem erhöhten Sturz- und Verletzungsrisiko beiträgt. Vor Etablierung oder Erweiterung einer blutdrucksenkenden Medikation wird eine Blutdruckmessung in Orthostase empfohlen. Die Blutdrucksenkung sollte langsam erfolgen, da mit dem Alter eine verzögerte Adaptation der Barorezeptoren und des sympathischen Systems assoziiert ist. Besondere Sorgfalt ist auf mögliche Interaktionen mit anderen Pharmaka und medizinischen Diagnosen zu legen. Unter Berücksichtigung von Komorbiditäten werden leitliniengerecht Kalziumantagonisten, Angiotensin-Converting-Enzyme(ACE)-Hemmer oder Angiotensin-II-Rezeptorblocker (ARB) empfohlen. Eine mögliche Ausnahme stellen β‑Blocker in der Initialtherapie dar (ohne spezifische β‑Blocker-Indikation), da diese Substanzgruppe den zentralen Blutdruck nur gering senkt. Medikamente, die zu einer orthostatischen Hypotonie beitragen (z. B. α‑Blocker), sind nicht erste Wahl.

### Antikoagulation bei Vorhofflimmern

Vorhofflimmern ist mit einer Prävalenz von 1–2 % der Bevölkerung eine häufige Erkrankung, Betroffene haben ein 4‑ bis 5fach erhöhtes Risiko für einen ischämischen SA. Mit dem Alter steigt die Prävalenz von VHF auf bis zu 15 % der 80-Jährigen. In einer australischen Studie war der Anteil kardioembolischer SA von 42 % vorwiegend durch VHF bedingt [9]. Durch **orale Antikoagulation**

orale Antikoagulation

(OAK) wird das SA-Risiko deutlich reduziert [10]. In den letzten Jahren wurden Vitamin-K-Antagonisten (VKA) weitgehend durch direkte orale Antikoagulanzien (DOAK) abgelöst. Die DOAK sind im Hinblick auf klinische Wirksamkeit und Sicherheitsprofil den VKA überlegen. Die Zulassungsstudien schlossen repräsentative Zahlen von Patient*innen mit relevanten Komorbiditäten ein; Post-Marketing-Analysen zeigen ähnliche Ergebnisse wie die Zulassungsstudien: Die DOAK sind auch im Alter wirksam und sicher [10]. Hohe Punktzahlen in Risiko-Scores wie CHA_2_DS_2_VASc („*c*ongestive heart failure“ [Herzinsuffizienz], *H*ypertension, *A*lter (>75 Jahre), *D*iabetes mellitus, „*s*troke“/transitorische ischämische Attacke [TIA], „*v*ascular disease“ (z. B. peripheren arteriellen Verschlusskrankheit [pAVK], vorangegangener Herzinfarkt), *A*lter (65 bis 74 Jahre), „*s*ex *c*ategory [weibliches Geschlecht]) bedingen jenseits des 75. Lebensjahres beinahe obligat eine Indikation für die OAK.

Ein häufiges Argument gegen eine OAK ist das Sturzrisiko alter Menschen, allerdings braucht es beinahe 300 Stürze, bevor das Risiko einer Blutung gegenüber dem eines SA überwiegt [11].

#### Merke

Das Sturzrisiko wird bei der Indikationsstellung zur OAK häufig überschätzt.

Die Kombination mit Thrombozytenfunktionshemmern (TFH), wie z. B. nach Gefäß-Stenting erforderlich, erhöht das Blutungsrisiko deutlich, sodass diese Perioden möglichst kurz gehalten werden sollten [12]. Vor Etablieren einer OAK ist ein **geriatrisches Assessment**

geriatrisches Assessment

sinnvoll, um potenzielle Risiken der Therapie hintanzuhalten: Besonders ist auf Mobilität, Sturzrisiko sowie kognitive und sensorische Fähigkeiten zu achten.

### Hypercholesterinämie

Der Zusammenhang zwischen hohen Werten des **Low-Density-Lipoprotein-Cholesterins**

Low-Density-Lipoprotein-Cholesterins

und SA ist gut belegt; die Datenlage für alte Menschen ist allerdings nicht eindeutig: Die meisten Erkenntnisse zum Einsatz von **Statinen**

Statinen

zur Primärprävention jenseits des 75. Lebensjahres stammen aus Subanalysen größerer Studien. Eine aktuelle Metaanalyse zum Einsatz von Statinen zur Primärprävention bei älteren Menschen umfasste 815.667 Patienten ohne klinisch manifeste Atherosklerose. Die Statintherapie war mit einem signifikant niedrigeren Risiko für Gesamtmortalität, kardiovaskulären Tod, SA und Myokardinfarkt assoziiert; die Reduktion der Gesamtmortalität blieb auch bei Personen > 75 Jahre signifikant [13]. Die aktuellen Leitlinien der European Society of Cardiology (ESC), der Heart Association (AHA) und des American College of Cardiology (ACC) enthalten keine eindeutigen Empfehlungen zum Einsatz von Statinen zur Primärprävention bei Personen > 75 Jahre ohne manifeste kardiovaskuläre Ereignisse [14]. Diese Leitlinien raten bei der Verschreibung von Statinen im Alter zu Vorsicht, besonders bei hohen Dosen, die das Nebenwirkungsrisiko erhöhen und die Lebensqualität verringern können, ohne die Lebenserwartung zu steigern.

### Thrombozytenfunktionshemmer

Aktuell werden TFH nicht zur Primärprävention von zerebro- oder kardiovaskulären Ereignissen empfohlen. Dies gilt insbesondere jenseits des 70. Lebensjahres; hier verschiebt sich ein möglicher Nutzen in Richtung des Risikos von **Blutungskomplikationen**

Blutungskomplikationen

[15].

#### Merke

Thrombozytenfunktionshemmer sind bei alten Menschen in der Primärprävention nicht indiziert.

### Diabetes mellitus und Hyperglykämie

Eine intensive Behandlung der Hyperglykämie mit konventionellen Medikamenten bei Patienten mit Diabetes reduziert das Risiko mikrovaskulärer Komplikationen, nicht aber das Risiko eines SA [16]. **Natriumglucose-Kotransporter-2-Hemmer**

Natriumglucose-Kotransporter-2-Hemmer

(SGLT2-Hemmer) senken das Risiko für schwerwiegende vaskuläre Ereignisse, einschließlich SA, im Vergleich zu Placebo [17]. Unter Glucagon-like-Peptide-1(GLP-1)-Rezeptor-Agonisten wurde eine Reduktion der Häufigkeit vaskulärer Ereignisse gezeigt, eine Reduktion von SA wurde aber nur für **Semaglutid**

Semaglutid

nachgewiesen [18]. Die Erfahrungen mit Patient*innen ≥ 75 Jahre sind begrenzt; die bei jüngeren Menschen oft erwünschte Reduktion des Körpergewichtes ist in Alterskollektiven kritisch zu sehen. Zu den Zielwerten der Blutzuckereinstellung wird im Abschn. „Sekundärprävention“ Stellung genommen“.

### Lebensstil

Es ist in jedem Alter sinnvoll, einen **Nikotinabusus**

Nikotinabusus

einzustellen. Die Empfehlungen zu **körperlicher Betätigung**

körperlicher Betätigung

und **bewusster Ernährung**

bewusster Ernährung

(z. B. mediterrane Diät, Dietary-Approaches-to-Stop-Hypertension[DASH]-Diät) können im Alter umgesetzt werden und gelten auch für die Sekundärprävention [3]. Eine **Grippe-Impfung**

Grippe-Impfung

kann das Risiko vaskulärer Ereignisse, einschließlich SA, reduzieren [19].

## Sekundärprävention

Kontrolle von Arterioskleroserisikofaktoren, Nikotinkarenz, antithrombotische Therapie und ggf. Intervention an den Karotiden sind Ansatzpunkte nach ischämischen SA; nach hämorrhagischen SA liegt der Schwerpunkt auf Blutdrucksenkung und Nikotinkarenz.

### Hypertonie

Eine konsequente Blutdruckkontrolle reduziert das SA-Rezidivrisiko um etwa 30 %. Die PROGRESS-Studie legt nahe, dass eine Blutdrucksenkung (mit Perindopril) auch bei nach WHO-Kriterien normalem Blutdruck positive Auswirkungen auf das Rezidivrisiko hat: Werte des systolischen Blutdrucks von 130–139 mm Hg zeigten das niedrigste Risiko [20]. Das Durchschnittsalter betrug 64 Jahre; die Risikoreduktion zeigte sich relativ unabhängig vom Lebensalter. In der SPS3-Studie war ein Wert des systolischen Blutdrucks < 130 mm Hg mit einem um 20 % geringeren SA-Risiko assoziiert als ein Wert von 130–149 mm Hg (statistisch allerdings nicht signifikant). Das Risiko für intrakranielle Blutungen war dagegen um 60 % geringer. Nach lakunärem Infarkt erscheinen Werte des systolischen Blutdrucks ≤ 130 mm Hg optimal. Das Durchschnittsalter in dieser Studie betrug 63 Jahre, die positiven Effekte waren jenseits des 65. Lebensjahres geringer ausgeprägt [21]. Alte Patient*innen haben neben einem hohen Risiko für SA auch ein hohes Risiko für andere kardiovaskuläre Ereignisse, was den Stellenwert adäquater Blutdruckkontrolle unterstreicht [22]. Die Auswahl der antihypertensiven Substanzen folgt aktuellen Leitlinien und berücksichtigt Nebenwirkungen und Begleiterkrankungen (s. Abschn. „Primärprävention, Hypertonie“).

#### Merke

Eine antihypertensive Therapie reduziert auch das Risiko intrakranieller Blutungen.

### Cholesterinsenkung

Als erste Studie zeigte SPARCL einen Effekt aggressiver Cholesterinsenkung auf den primären Endpunkt SA. Das SA-Risiko wurde mit **Atorvastatin**

Atorvastatin,

80 mg/Tag, gegenüber Placebo signifikant gesenkt: 11,2 % vs. 13,1 %. Das Durchschnittsalter der Teilnehmenden in dieser Studie betrug 63 Jahre; der Nutzen der Therapie war jenseits des 65. Lebensjahres geringer ausgeprägt. Metaanalysen und epidemiologische Daten belegen die Wirkung der Cholesterinsenkung in der Sekundärprävention auch bei alten Patient*innen: Die Risikoreduktion für tödliche kardiovaskuläre Ereignisse durch eine Cholesterinsenkung von 1 mmol/l (38,67 mg/dl) beträgt zwischen 40 und 49 Jahren 50 %, beträgt aber für 70- bis 89-Jährige respektable 17 % [23]. Auch bei > 75-Jährigen führt eine lipidsenkende Therapie zu einer signifikanten Senkung von koronaren Ereignissen (10,6 % vs. 12,8 % in der Kontrollgruppe) und Major Vascular Events (16,8 % vs. 19,7 %; [24]).

Die Zielwerte für alte Patient*innen sind nicht einheitlich definiert: Die SA-Patient*innen werden nach den Leitlinien von ESC/European Atherosclerosis Society (EAS) zur „Very-high-risk“-Gruppe mit einem LDL-C-Zielwert < 55 mg/dl gezählt. Die Leitlinien der Deutschen Gesellschaft für Neurologie empfehlen einen LDL-Zielwert < 70 mg/dl, alternativ kann eine Reduktion > 50 % des Ausgangswerts angestrebt werden [25]. Diese Zielwerte erfordern meist den Einsatz von hochpotenten Statinen. Da Frailty, Multimorbidität und Polymedikation das Risiko unerwünschter Arzneimittelwirkungen erhöhen, muss die Balance zwischen Risiko und Nutzen der Lipidsenkung individuell abgewogen werden. Im Hinblick auf die im Alter erhöhte Sturzgefahr ist eine Beeinträchtigung der Muskelfunktionen durch Statine zu beachten. Bei hochbetagten Patienten mit geringer Lebenserwartung (< 1 Jahr) ist die Datenlage für eine klare Empfehlung nicht ausreichend.

### Antithrombotische Therapie

#### Orale Antikoagulation

Nach einem SA sind alte Menschen mit VHF besonders gefährdet, ein Rezidiv zu erleiden, daher sollte die OAK allen geeigneten Patient*innen angeboten werden. Eine gepoolte Analyse von 2 randomisierten Studien zeigte eine relative Risikoreduktion von 64 % für die OAK vs. Placebo [26]. Eine Metaanalyse ergab keinen signifikanten Unterschied zwischen DOAK und VKA in der Verhinderung von SA oder systemischer Embolie, allerdings eine signifikante Reduktion von intrakraniellen Blutungen und Tod unter DOAK („relative risk“ [RR] 0,43; 95 %-Konfidenzintervall [95 %-KI] 0,29–0,64, [26]). Dies gilt auch für Patient*innen > 75 Jahre, gebrechliche alte Menschen sind aber auch in diesen Studien unterrepräsentiert. Ausschlussgründe für eine OAK gibt es wenige; anzuführen sind die Anamnese einer hämorrhagischen Diathese oder einer rezenten gastrointestinalen Blutung. Bei erhöhtem Sturzrisiko sollte eine sorgfältige Abklärung möglicher Ursachen erfolgen. Dazu ist ein geriatrisches Mobilitätsassessment erforderlich. Kraft- und Gleichgewichtstraining, die Verordnung von Hilfsmitteln und Anpassung der Umgebung sind Therapieoptionen.

##### Merke

Ein geriatrisches Assessment ist zur Abschätzung des Sturzrisikos indiziert.

Der optimale Zeitpunkt für das Starten einer OAK nach SA richtet sich der **Infarktgröße**

Infarktgröße

und anderen Begleitfaktoren. Die ELAN-Studie zeigte, dass eine frühe Antikoagulation (< 48 h nach leichtem/mittelschweren SA, Tage 6 und 7 nach schwerem SA), verglichen mit einem späteren Beginn, sicher ist und das Risiko für einen erneuten SA reduziert, ohne das Blutungsrisiko zu erhöhen [27].

#### Thrombozytenfunktionshemmer

Eine Therapie mit TFH ist bei allen nichtkardioembolischen und atherosklerotisch bedingten SA indiziert. Für die Sekundärprophylaxe fand sich unter **Acetylsalicylsäure**

Acetylsalicylsäure

(ASS) eine absolute Reduktion des kombinierten Endpunkts Herzinfarkt, SA und vaskulär bedingter Tod (6,7 % vs. 8,2 % pro Jahr, *p* < 0,0001) mit einem nichtsignifikanten Anstieg hämorrhagischer SA sowie einer Reduktion aller SA (ischämisch und hämorrhagisch; 2,1 % vs. 2,5 % pro Jahr, *p* = 0,002, [28]). Studien mit ASS legen nahe, dass die Behandlung möglichst rasch nach dem Ereignis begonnen werden sollte. Die Vorteile der früheren Sekundärprävention nach einer TIA oder ischämischem SA sind innerhalb der ersten ein bis 2 Wochen ähnlich wie innerhalb der nachfolgenden 50 Wochen. Metaanalysen zeigen keine Interaktion mit dem Alter [29].

##### Merke

Eine Therapie mit TFH sollte möglichst rasch nach dem Ereignis begonnen werden.

Nach einer TIA mit hohem Rezidivrisiko oder leichtem Schlaganfall ist für die ersten 21 Tage die Kombination von ASS mit **Clopidogrel**

Clopidogrel

vorteilhaft [30]. Diese Kombination ist in der Langzeitanwendung aufgrund einer erhöhten Inzidenz von Blutungen nicht etabliert. Nach einer Thrombolyse oder Thrombektomie wird mit der TFH nach Exklusion hämorrhagischer Komplikationen begonnen. Dies erfolgt mithilfe einer zerebralen bildgebenden Untersuchung; eine Computertomographie gilt als ausreichend.

Es gibt keine klare Evidenz für eine unterschiedliche Wirksamkeit verschiedener TFH bei alten Menschen. Damit folgt die Auswahl der Substanzen einer individualisierten Entscheidung, die auf den Präferenzen von Patient*innen und Ärzt*innen, Begleitmedikation und Nebenwirkungsprofil basiert. Für ASS mehren sich Hinweise, dass das Komplikationsrisiko mit dem Alter steigt: Eine prospektive populationsbasierte Kohortenstudie untersuchte über 10 Jahre Auftreten und Outcome von Blutungskomplikationen bei 3166 Patienten, die nach erstmaliger TIA, ischämischem SA oder Myokardinfarkt mit TFH (meist ASS) behandelt wurden. Im Lebensalter ≥ 75 Jahre fand sich eine Zunahme schwerer („hazard ratio“ [HR] 3,10; 95 %-KI 2,27–4,24, *p* < 0,0001) und fataler Blutungen (HR 5,53; 95 %-KI 2,65–11,54, *p* < 0,0001), wobei behindernde bis fatale Blutungen im oberen Gastrointestinaltrakt mit einem absoluten Risiko von 9,15 (95 %-KI 6,67–12,24) pro 1000 Patientenjahren häufiger waren als behindernde und fatale intrazerebrale Blutungen [31]. Dennoch wird bei Fehlen gastrointestinaler Probleme das routinemäßige Verabreichen eines Protonenpumpenhemmers unter ASS nicht empfohlen [25].

### Diabetes mellitus

Für die Blutzuckerzielwerte, die bei Diabetes mellitus nach ischämischem SA angestrebt werden sollen, liegen nur Ergebnisse vor aus Subgruppenanalysen randomisierter Studien, in denen geriatrische Patient*innen nicht ausreichend berücksichtigt wurden. Bei alten Patient*innen mit Diabetes mellitus ohne funktionelle Einschränkungen wird ein Zielwert des HbA_1c_ ≤ 7,5 %, bei leichten funktionellen Einschränkungen (Multimorbidität, kognitive Einbußen, geriatrische Syndrome) ein Zielwert des HbA_1c_ ≤ 8,0 % und bei ausgeprägter funktioneller Abhängigkeit (Multimorbidität, geriatrische Syndrome, deutliche funktionelle oder kognitive Defizite, limitierte Lebenserwartung) ein Zielwert des HbA_1c_ < 8,5 % empfohlen. Diese weniger strengen Zielwerte sind begründet durch das Vermeiden von Hypoglykämien, die zu einem erhöhten Risiko für kardiovaskuläre Ereignisse und Demenz beitragen.

### Karotisintervention

**Endarteriektomie**

Endarteriektomie

und endovaskuläre Verfahren sind in der Primär- und Sekundärprävention von SA hinlänglich untersucht. Ob die Sanierung einer **asymptomatischen Karotisstenose**

asymptomatischen Karotisstenose

indiziert ist, kann nicht klar beantwortet werden, da es seit der Durchführung der zugrunde liegenden Vergleichsstudien zu einem deutlichen Fortschritt in der konservativen Therapie gekommen ist. Bei **symptomatischer Karotisstenose**

symptomatischer Karotisstenose

wird die Sanierung ab einem Stenosegrad von ≥ 70 % empfohlen, bei Stenosen von 50–69 % erfolgt ein Abwägen anhand des **individuellen Interventionsrisikos**

individuellen Interventionsrisikos.

Eine Metaanalyse der ECST- und NASCET-Studien zeigte, dass höheres Lebensalter keine Kontraindikation für eine Operation darstellt, aufgrund des erhöhten absoluten Risikos könnte sogar ein größerer Netto-Benefit vorliegen [32]. Die Endarteriektomie dürfte jenseits des 70. Lebensjahres überlegen sein, ebenso wie die Intervention innerhalb der ersten 2 Wochen nach dem SA [33]. Nach TIA oder Minor Stroke ist eine rasche Abklärung einer Karotisstenose erforderlich. **Stenting**

Stenting

ist eine Option für jene, die einer Operation nicht zustimmen oder für eine Endarteriektomie nicht geeignet sind.

## Fazit für die Praxis

- Bei fitten alten Menschen gelten die Leitlinien zu Primär- und Sekundärprävention von Schlaganfall vollinhaltlich: Das zukünftige Schlaganfallrisiko kann deutlich reduziert werden; daher ist es sinnvoll, Betroffene von diesen Therapieoptionen zu überzeugen.
- Bei gebrechlichen alten Menschen ist die Datenbasis weniger verlässlich. Hier sind individualisierte Therapieentscheidungen erforderlich, um möglichst eine hohe Wirksamkeit sicherzustellen.
- Besonders zu beachten sind in diesem Kontext Blutungs- und Sturzrisiko sowie die Handhabbarkeit der Therapie. Ein geriatrisches Assessment stellt eine wesentliche Entscheidungsgrundlage dar.

## CME-Fragebogen

Wieviel Prozent aller Schlaganfälle treten jenseits des 80. Lebensjahres auf?

Etwa 5 %

Etwa 10 %

Etwa 30 %

Etwa 70 %

Etwa 90 %

Ihre 81-jährige Patientin hat eine Hypercholesterinämie mit einem Low-Density-Lipoprotein[LDL]-Wert von 160 mg/dL, ihr systolischer Blutdruck beträgt unter blutdrucksenkender Medikation im Mittel um 130 mm Hg. Seit sie vor 5 Jahren einen leichten Schlaganfall erlitten hat, ist sie auf ASS eingestellt. In den letzten 3 Monaten ist sie zweimal gestürzt, daher indizieren Sie ein Sturz-Assessment. Sollte an der Medikation etwas geändert werden?

Die Medikation sollte unverändert bleiben.

Der Beginn einer Statintherapie sollte mit der Patientin besprochen werden.

Die Blutdruckmedikation sollte angepasst werden, um den systolischen Wert unter 120 mm Hg zu senken.

Unter ASS ist die Gabe eines Protonenpumpenhemmers (PPI) obligat.

ASS soll auf Grund der Stürze und des damit verbundenen Blutungsrisikos abgesetzt werden.

Ein 81-jähriger Mann, körperlich und geistig fit, erleidet einen Schlaganfall mit leichtgradiger Hemiparese, in der Computertomographie kommt ein lakunärer Infarkt zur Darstellung. Bis auf eine medikamentös behandelte Hypertonie und eine benigne Prostatahyperplasie sind keine aktuellen Erkrankungen bekannt. Die in der Klinik gemessenen Blutdruckwerte liegen systolisch zwischen 145 und 165 mm Hg. Wie sollte nun weiter verfahren werden?

Der Blutdruck kann so belassen werden, da im Alter die Grenzwerte höher sind.

Der Blutdruck sollte nicht unter 140 mm Hg gesenkt werden, da bei niedrigeren Zielwerten das Sturzrisiko steigt.

Der Blutdruck sollte auf etwa 140 mm Hg gesenkt werden, weil es sich um einen lakunären Infarkt handelt.

Der Blutdruck sollte auf ≤ 130 mm Hg gesenkt werden, da dies laut Studien bei lakunären Infarkten der optimale Zielwert ist.

Die aktuelle Blutdruckmedikation sollte frühestens in 3 Monaten verändert werden, da in den ersten Wochen nach einem Schlaganfall die Blutdruckwerte sehr stark schwanken.

Die Prävalenz von Vorhofflimmern steigt mit dem Alter. Wie hoch ist sie bei 80-Jährigen?

3 %

5 %

10 %

15 %

30 %

Ihr 78-jähriger Patient hat einen leichten ischämischen Schlaganfall erlitten. Auf Grund von Vorhofflimmern soll eine orale Antikoagulation angesetzt werden. Ab welchem Zeitpunkt nach dem Schlaganfall kann diese Medikation mit optimalem Nutzen-Risikoverhältnis begonnen werden?

Innerhalb von 48 Stunden

Nach 10 Tagen

Nach 2 Wochen

Nach 4 Wochen

Nach 2 Monaten

Was ist zu beachten, wenn bei einer hochaltrigen Patientin nach einer TIA (transitorische ischämische Attacke) mit hohem Rezidivrisiko eine Therapie mit Thrombozytenfunktionshemmern indiziert ist?

Ein Protonenpumpenhemmer sollte dauerhaft dazugegeben werden.

Ein Protonenpumpenhemmer sollte in den ersten 2 Monaten dazugegeben werden.

ASS sollte dauerhaft mit Clopidogrel kombiniert werden.

ASS sollte in den ersten 3 Wochen mit Clopidogrel kombiniert werden.

ASS sollte frühestens 3 Wochen nach der TIA begonnen werden.

Eine 83-jährige Patientin mit Diabetes mellitus hat einen mittelschweren ischämischen Schlaganfall erlitten. Sie ist multimorbide und hat leichte kognitive Einbußen. Welcher Ziel-HbA1c-Wert wird in diesem Fall angestrebt?

≤ 6,5 %

≤ 7 %

≤ 7,5 %

≤ 8 %

≤ 8,5 %

Ein fitter 75-jähriger Mann erleidet eine TIA (transitorische ischämische Attacke) im Stromgebiet der linken Arteria cerebri media. Im Rahmen der Ursachenabklärung wird ipsilateral eine Stenose der Arteria carotis interna festgestellt. Ab welchem Stenosegrad wird hier eine Sanierung empfohlen?

≥ 40 %

≥ 50 %

≥ 60 %

≥ 70 %

≥ 80 %

Sie klären einen 84-jährigen Patienten nach einem ischämischen Schlaganfall bei Vorhofflimmern (VHF) über Nutzen und Risiko einer oralen Antikoagulation (OAK) auf. Die neurologische Symptomatik hat sich weitgehend zurückgebildet. Welches Argument trifft zu?

Nach einem Schlaganfall sind hochaltrige Menschen mit VHF kaum gefährdet, einen weiteren Schlaganfall zu erleiden.

Eine gepoolte Analyse zweier randomisierter Studien zeigte eine relative Risikoreduktion von 64 % für die OAK gegenüber Placebo.

Für direkte orale Antikoagulanzien (DOAK) zeigte sich in Vergleichsstudien zu Vitamin-K-Antagonisten (VKA) ein erhöhtes Risiko für intrakranielle Blutungen und Tod.

Für DOAK liegen Sicherheitsdaten aus Studien vor, die ausschließlich Patient*innen jenseits des 80. Lebensjahres untersuchten.

Bei VHF soll die OAK frühestens 1 Monat nach dem Schlaganfall gestartet werden.

Bluthochdruck ist ein Hauptrisikofaktor für Schlaganfälle, randomisierte kontrollierte Studien zeigten eine deutliche Risikoreduktion durch Therapie. Ab welchem systolischen Wert wird in der Primärprävention ein Therapiebeginn bei weitgehend gesunden alten Menschen empfohlen?

> 120 mm Hg

> 125 mm Hg

> 130 mm Hg

> 140 mm Hg

> 150 mm Hg
